# Association of Smoking-Related Knowledge, Attitude, and Practices (KAP) with Nutritional Status and Diet Quality: A Cross-Sectional Study in China

**DOI:** 10.1155/2019/5897478

**Published:** 2019-08-21

**Authors:** Ijaz ul Haq, Yu Liu, Min Liu, Haifeng Xu, Hui Wang, Chunlan Liu, Falak Zeb, Pan Jiang, Xiaoyue Wu, Yuanrui Tian, Mengxia Li, Qun Li, Jun Fu, Chong Shen, Qing Feng

**Affiliations:** ^1^Department of Nutrition and Food Hygiene, School of Public Health, Nanjing Medical University, Nanjing 211166, China; ^2^Center for Disease Control and Prevention of Jurong City, Jurong 212402, China; ^3^Department of Epidemiology, School of Public Health, Nanjing Medical University, Nanjing 211166, China; ^4^Department of Clinical Nutrition, Affiliated Yifu Hospital, Nanjing Medical University, Nanjing 211166, China

## Abstract

**Background:**

Smoking is the second leading cause of death. Limited studies are available about smoking and overall diet quality. The current study was aimed at finding an association of s-KAP (smoking-related knowledge, attitude, and practices) with nutritional status and diet quality.

**Methodology:**

The current study was a cross-sectional community-based study conducted in Jurong city, China. Validated questionnaires were used for the collection of data regarding s-KAP and dietary intake. Correlation and multivariate linear regression analysis were used for the association of s-KAP scores with diet quality scores and nutritional status.

**Results:**

The total numbers of participants were 7998 with a mean age of 59.3±11.4 years, including 38.5% males and 41.5% females. s-KAP scores were categorized into two groups, i.e., High s-KAP group and low s-KAP group. The High s-KAP group had significantly higher (*P<0.05*) diet scores and BMI but lower (*P<0.05*) WC (waist circumference) and WHR (waist to hip ratio) than the Low s-KAP group. Independent positive association (*P<0.05*) of s-KAP scores with diet scores was observed after the adjustment for age, gender, physical activity, alcohol consumptions, monthly income, and anthropometric measures (BMI, WC, and WHR). Similarly, smoking was positively associated (*P<0.05*) with diet scores after adjustment for covariates.

**Conclusion:**

In conclusion, the higher s-KAP scores indicated more knowledge regarding the harmful consequences of the smoking outcomes, positive attitude, less smoking practices, and having a good plan to quit smoking. Individuals with high s-KAP scores had good diet quality and lower adiposity measures. Furthermore, s-KAP scores and smoking status were having an independent positive association with diet scores.

## 1. Introduction

China is the largest tobacco consumer, consisting of 300 million current smokers. In 2010, 28.1% of Chinese adults, including 52.9% of men and 2.4% of women were smokers [1]. The second leading cause of death and a significant risk factor of mortality in China are smoking [2]. Also, smoking is an independent risk factor for chronic diseases like diabetes, cardiovascular diseases, chronic respiratory diseases, chronic kidney disease, and various types of cancers [3].

Chronic diseases like cancer, diabetes, and cardiovascular diseases, associated with smoking are also modifiable by diet. Poor diet quality can increase the risk of mortality [4] and may exacerbate the risk of cancer and coronary heart diseases [5] while healthy diet and lifestyle (including no-smoking, low alcohol consumption, and proper physical activity) could prevent cardiometabolic diseases [6]. Diet quality might be influenced by nutrition knowledge, as nutritional knowledge is an influencing factor for the selection of a healthy diet [7]. Nutrition awareness has been found to have a direct effect on diet quality, as indicated by diet diversity, energy density, and adequacy to achieve dietary recommendations [8]. Higher nutrition knowledge is positively associated with diet quality and lower blood pressure[9].Earlier, a study showed that smokers consume lower quality of diet as their essential nutrients intake is lesser as compared to nonsmokers [10]. Moreover, Alkerwi et al. found an inverse relationship between the intensity of tobacco consumption and overall diet quality in people from Luxembourg [5]. It might be possible that people who have good smoking KAP scores also have good nutrition knowledge. Thus, they are likely to have a good diet quality.

Specific intervention is needed, which can support the community to take care regarding their own health as well as their families' health. Hence, it seems essential to understand individuals' smoking-related knowledge, attitude, and practices (s-KAP) along with diet consumption and nutritional status for better interventions. Previously, some studies explored tobacco-related KAP in a specific population or with particular objectives in China. For example, s-KAP was investigated in nurses who had some knowledge about the impact of tobacco use on human health, but they seldom practiced smoking cessation interventions [11]. Another study reported that a high smoking rate and insufficient knowledge regarding the impact of smoking on health among male health care providers might be a barrier to tobacco control efforts [12]. One study in young male adult found that smokers with higher education had better knowledge and positive attitude towards smoking but poor behavioral outcomes [13].

Previously, the relation between individual specific essential nutrients (macro and micronutrients) and food intake, including tobacco smoking status, have been described in the literature [10, 14]. However, the relationship between tobacco smoking and overall diet quality are rare. Recently, the focus of some epidemiological studies changed from single nutrients to overall dietary patterns scores [15–17]. Therefore, in the current study, we also focused on overall diet quality scores. The purpose of this study was to investigate the existing relationship of s-KAP scores with nutritional status and dietary scores among the respondents in China. Based on these investigations, the association between these variables could improve the public health messages aimed at smoking cessation and tobacco control and promote healthy dietary behaviors in this targeted population of a developing country.

## 2. Methods

### 2.1. Study Population

The study subjects in this cross-sectional survey were selected by multi-stage sampling method from October to November 2015 in Jurong city, Jiangsu Province, China. A total of 11151 subjects aged 18-97 years participated in the survey.

**Figure 1:**
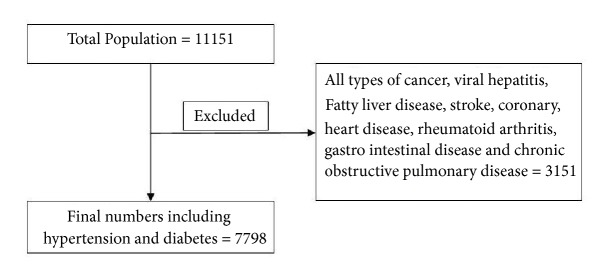
Total 11,151 individuals were investigated for this study. Individuals with chronic diseases (N=3153) were excluded. Finally, 7,798 individuals were included in this study.

Firstly, we randomly selected 8-10 villages in each township in a total of 13 townships and then randomly selected 100 subjects in each village. This study included the individuals with complete smoking and diet information according to the questionnaire and excluded the subjects with incomplete information of smoking and diet as well as with some chronic disease which might influence diet quality. The final numbers of participants for analysis were 7998 (Figure 1).

The study protocol was approved by the ethical review board of Nanjing Medical University, according to the Declaration of Helsinki. Written informed consent was obtained from each participant.

### 2.2. Data Collection

Data were collected by trained personnel, who were trained regarding techniques of interview, practical applications, tools of data collection, and area guidelines before the data collection.

A predesigned and pretested questionnaire was used for the collection of data from all participants. The standard survey was including age, gender, educational levels, smoking status, SHS status, s-KAP, drinking information, income levels, physical activity index level (PAI), and nutritional status based on anthropometric measurements.

#### 2.2.1. Smoking-Related KAP

*(1) Smoking*. Smoking status was divided into three categories, i.e., current smokers, occasional smokers, and nonsmokers.

*(2) Second-Hand Smoking (SHS)*. Second-hand smoking status was categorized into the seldom exposed group and exposed the group. The exposed group was defined as individuals who were exposed to second-hand smoking more than 15 minutes per day.

*(3) s-KAP Questionnaire and Scores*. The s-KAP questionnaire (supplementary Table 1) was adopted from the previous literature [13], consisting of three sections. Furthermore, for reliability, the Cronbach alpha of the questionnaire was more than 0.70. In the knowledge section, a total of eight questions were included regarding the relation of smoking (both active and passive) with diseases. In the attitude section, five items were included about the attitude of the participants towards smoking. Finally, in the practice section, four questions were included. The total maximum s-KAP scores were 20, including eight scores for knowledge, five scores for attitude, and seven scores for practices. Furthermore, s-KAP scores were divided into two groups, i.e., Low s-KAP and High s-KAP groups. The Low s-KAP group had respondents scored below the mean score while the High s-KAP group had respondents scored a mean score or above.

#### 2.2.2. Dietary Assessment and Diet Scores

A previously validated food frequency questionnaire was used for the dietary assessment [18]. The quantity of each food was reported in Liang (a Chinese unit for the measurement equal to 50 grams). FFQ consists of food groups, i.e., cereals (rice, wheat, and noodles), grains, eggs, poultry and meat, milk and milk products, vegetables, fruits, oil, fish and shrimps, salt and sugar. Diet quality scores were calculated for all individuals according to the previously described Chinese dietary guideline index scores (online supplementary file Table S2) according to the recommended energy intake food intake. Briefly, the CDGI components were including six adequacy components including grains, vegetables, fruits, nuts and soya bean products, milk & products, and seafood whereas four moderate products were included meat and poultry, oil, salt, and alcohol. For each component, 0-10 with the maximum total score of 100 scores was given [19].

#### 2.2.3. Anthropometry

Height was measured in cm to the nearest 0.1 cm with bare feet, and weight was measured in kg to the nearest 0.1 kg with light clothing. BMI was calculated as kg/m^2^ and categorized according to cutoffs for Chinese adults as underweight (<18.5), normal (18.5-23.9), overweight (24.0-27.9) and obese (≥28)[20]. The measuring tape was used to measure waist circumference (WC) in cm to the nearest 0.1 cm around the abdomen at the level of the umbilicus (belly button). Central obesity was defined as WC>90 cm for males and 80 cm for females [21]. WHR was calculated to find abdominal obesity. Abdominal obesity was defined as WHR of more than 0.94 for males and 0.80 for females [22]. BMI, WC, and WHR were referred to as nutritional status.

#### 2.2.4. Other Covariates

Educational levels were divided into less than middle schooling, middle schooling, high schooling, and college and above levels. Income level was reported in Chinese Yuan earning by each person per month and categorized into three groups, i.e., <2000, 2000-4000 and >4000. Smoking consisted of current smokers, occasional smokers, and never smokers. Drinking status was divided into drinkers and nondrinkers. PAI was calculated as the time spent in different activities multiplied by the assigned metabolic equivalents (MET) values: housework and walking= 3.0 MET, gardening or farming= 4.0 MET, home repairs = 4.5 MET, cycling and sports= 6.0 MET, and stair climbing=8.0 MET [23].

The covariates were selected according to the previous literature. Physical activity [24], socioeconomic position based on income [25], education [26], alcohol consumption [27], and nutritional status [28] are the direct influencing factors for diet quality. All models were adjusted for these covariates in multivariate analysis.

### 2.3. Statistical Analysis

Data were analyzed using IBM SPSS Statistics, Version 22.0 (IBM Corp, Armonk, NY, USA) and expressed as mean ± SD for normally distributed continuous data, median and interquartile range for not normally distributed continuous data, and frequency and percentages for categorical data. Independent t-tests and Mann Whitney tests were used for the comparison of continuous variables while chi-square tests were used for categorical variables. Independent t-tests were used to find the comparison of anthropometric measurements and diet scores between two groups of s-KAP scores. Spearman correlations were used for the relation of s-KAP scores with dietary intake and anthropometric measurements. Multiple linear regressions were used for the association of diet scores (dependent variable) with the s-KAP scores after adjustment of the covariates (age, gender, PAI, alcohol consumption, education levels, income, and anthropometric measurements including BMI, WC, and WHR). There was no issue of heteroskedasticity in our linear regression model because the plot of residuals showed an even envelope of residuals created in the linear regression plots for all models. As for multicollinearity, the tolerance score was more than 0.1, and the variance inflation factor (VIF score) was between 1 and 10 for all models. A two-tailed P value less than 0.5 were considered as statistically significant.

## 3. Results

### 3.1. General Characteristics of the Study Population

A total of 7998 respondents (61.5% females and 38.5% males) with mean age 59.3 years were included in this study (Table 1). Educational levels, smoking, drinking, BMI, central and abdominal obesity was significantly associated with gender (*P<0.05*). Females had significantly higher (*P*<0.01) s-KAP scores than male participants. Male's respondents had significantly higher (*P*<0.01) diet scores than females. Females had significantly higher (*P*<0.01) s-KAP scores than male participants. Male's respondents had significantly higher (*P*<0.01) diet scores than females.

### 3.2. Comparison of Smoking-Related KAP according to Diet Scores and Anthropometric Measurements

Diet scores and anthropometric measurements were compared between the low and high s-KAP groups (Table 2). Diet score of the High s-KAP group was significantly higher than the group with low s-KAP scores. Regarding weight status, BMI of Low s-KAP groups (24.8±3.7) was significantly higher (*P<0.05*) than the High s-KAP group (25.0±3.4). The WC of the Low s-KAP group was 82.5±9.7, significantly higher (*P<0.05*) than the High s-KAP group. Similarly, the WHR of the low s-KAP group (0.87±0.1) was significantly lower (*P<0.05*) than the other group. Although values of adiposity measures for both groups were much higher from the standard values. There was a significant difference between the diet scores and s-KAP scores of SHS and smoking groups (Supplementary Table 3, P<0.01).

### 3.3. Dietary Intake and Correlation Analysis

Dietary intake of respondents according to s-KAP groups has shown in online supplementary file Table S4. Cereals intake, alcohol consumption, sugar, and salt intake were higher (*P<0.05*) in the low s-KAP group. Whole grains, potato, seafood, nuts and soy products, poultry and meat, milk and products, and fruits intake of low s-KAP were significantly lower (*P<0.05*) than the high s-KAP group.

The correlation of the food group's daily intake with s-KAP scores has been shown in online supplementary file Table S5. For the whole population, a negative correlation (*P*<0.01) of s-KAP scores was observed with cereals (rice, wheat flour, noodles) intake (r=-0.178) and alcohol intake (r=-0.161). While a positive correlation (*P<0.01*) with whole grain (r=0.157), potato (r=0.125), seafood (r=0.1), and milk and products (r=0.157) with s-KAP scores was observed.

### 3.4. Association of s-KAP Scores with Nutritional Status (BMI, WC, and WHR)

**Figure 2:**
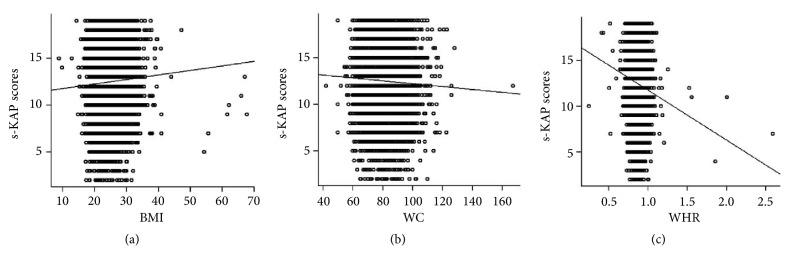
Association of nutritional status with s-KAP scores (a) Relation between BMI with s-KAP scores (r=0.050, P<0.05) (b) Relation between WC with s-KAP scores (r=-0.047, P<0.05) (c) Relation between WHR with s-KAP scores (r=-0.103, P<0.05).

Figures 2(a), 2(b), and 2(c) show the association of s-KAP scores with nutritional status. As for BMI, the trend of the association of s-KAP was positive r=0.050, while a negative (*P*<0.05) with WC (r=-0.047) and WHR (r=-0.103). This association shows a week association between s-KAP scores and nutritional status. Through linear regression, s-KAP was having no significant association with BMI, WC, and WHR after adjustment for covariates (Supplementary Table S6).

### 3.5. Association of s-KAP Scores, Smoking, and SHS with Diet Scores

Multivariate models confirmed an independent association of s-KAP scores (P<0.01) with diet quality scores in all three models after the adjustment of other covariates including age, gender, physical activity index, alcohol consumptions, educational levels, income, BMI, WC and WHR(Table 3). In stratification analysis, s-KAP scores were also having an independent association (P<0.01) with diet quality scores in with and without hypertension and diabetes in all three models. As for smoking status, all three models confirmed smoking status as an independent influencing factor of diet quality (Supplementary Table 7). In stratification analysis for hypertension and smoking, smoking status was significantly associated with diet (P<0.05). Second-hand smoking did not influence diet quality in multivariate models (Supplementary Table 8, P>0.05).

## 4. Discussion

The current study demonstrated that s-KAP scores had an independent positive association with diet scores after the adjustment of other covariates. Respondents in low s-KAP scores group had lower diet scores; higher WC and WHR. The respondents in the high s-KAP scores group had a good dietary intake. Furthermore, smoking was having an independent negative associated with diet quality, while SHS was not significantly associated with diet quality. We did not observe the significant association between nutritional status based on BMI, WC, and WHR with diet quality. Up to our best knowledge, this is the first study which determined the association of s-KAP sores with nutritional status based on anthropometric measurements and diet scores in the Chinese population.

The higher s-KAP scores indicated more knowledge regarding the harmful consequences of the smoking outcomes, positive attitude, and a good plan to quit smoking and less smoking practices. High smoking-related knowledge or awareness regarding the health risks of smoking might have a little impact on healthy behavior outcomes [29] because this knowledge must need to merge with a positive attitude to minimize unsafe and unhealthy smoking practices, which lead to smoking cessation. Likely, nonsmoker individuals are keen to know the consequences of the smoking outcomes; also, they put their positive attitude into action and do not practice smoking. The positive attitude may also develop assertive behavior in nonsmokers, i.e., asking smokers not to smoke help in promoting a smoke-free environment as well [30]. This heterogeneity between nonsmokers and smokers is significant for tobacco control. Earlier, a study found systematic heterogeneity between nonsmoker and smokers about smoking restrictions [31].

Furthermore, due to heterogeneity in behavior, smokers, as compared to nonsmokers, are less likely to acknowledge the consequences of smoking [32]. Earlier smoking cessation has more significant health benefits and can be observed even decades after quitting of smoking [33]. Although it is not necessary that smoking-related knowledge and attitude could translate into quitting smoking or less smoking practices [13].

Individuals who are aware more about their health are less likely to indulge in unhealthy behavior like smoking practice. Following a healthy lifestyle can decrease smoking behavior and practices [34]. We found a negative correlation of s-KAP scores with WHR but week positive correlation with BMI and week negative with WC. Although in multivariate regression analysis, we did not observe significant association between s-KAP scores and nutritional status. We divided the s-KAP into two subgroups, i.e., low s-KAP group and high s-KAP group. Also, WC and WHR of the Low s-KAP groups were higher than high s-KAP groups. Earlier, it has been documented that higher adiposity measurements influence smoking behavior [35]. Another study also concluded that an increase in cigarette consumption leads to abdominal obesity in healthy women [36]. In literature, BMI has been shown to positively associated with smoking intensity. There might be other influencing factors, including alcohol consumptions, physical inactivity, and unhealthy diet [37].

For better health, balanced and healthy diets, including a variety of food groups are acceptable for individuals (US Department of Health and Human Services and US Department of Agriculture, 2005). Good dietary habits can predict a healthy diet. Dietary habits could be a critical factor in determining health and nutritional status [38]. To investigate the difference in dietary intake among the respondents, we compared the dietary intake of low and high s-KAP groups. The more comprehensive assessment of our study demonstrated that, compared to the dietary intake of the Low s-KAP group, the dietary intake reported by the high s-KAP group was better. The food of low s-KAP had lower consumption of whole grains, seafood, milk and products, nuts and soy products, fruits, and high intake of salt and cereals of high carbohydrate contents. Also, the overall diet quality of High s-KAP group was better than Low s-KAP group. These are the novel findings discussed in the present study.

To investigate relationships between s-KAP scores and diet quality, we examined the overall scores of smoking-related to knowledge, attitude, and practices with overall diet quality among the respondents. In general, smoking KAP scores were positively associated with diet qualityscores. Individuals who had higher KAP scores are likely to engage in healthy eating behaviors, which include good quality of diet. These are the novel findings and never investigated before.

Furthermore, the results from the current study also provide an update that smoking negatively affects overall diet quality, which predicts inadequate dietary intake among the smoking population. In literature, it is reported that smokers consume lower quality of diet as their essential nutrients intake is lesser as compared to nonsmokers [10]. Smokers are less likely to engage in healthy lifestyle behavior, i.e. following unhealthy dietary practices and physical activity [39]. It is very challenging to evaluate specific nutrients for the translation into intervention for smokers. Therefore identification of overall diet quality may better allow for identification strategies to target for interventions [40]. Our findings were consistent with a previous result who found that smoking status is inversely associated with diet quality in a population based on samples from Luxembourg [5].

Strengths of the study include a relatively large number of sample sizes, comprehensive information, and validated questionnaires. However, there were several limitations which also need to be accounted for. First, the study design was cross-sectional, which may reduce the researcher's ability to make direct causality of the association with variables. Second, dietary information collected by FFQ over one year may have recalling biases. Third, we calculated the amount of food in Liang (50 grams), may not be the exact estimate of the food consumed by the respondents. Last, the observed correlation of s-KAP, nutritional status, and diet scores needs further confirmation in a cohort study. Nevertheless, the current study is the first study which investigated the relation of s-KAP scores with nutritional status and dietary scores in the Chinese population.

## 5. Conclusion

In conclusion, the diet quality scores and nutritional status (WC and WHR) of the high s-KAP group were better than the low s-KAP group. Furthermore, s-KAP scores were having an independent positive association with diet scores while a negative correlation with WHR. Smoking in Chinese population also affects their diet quality. Individuals with good quality food had lower adiposity measures along with good smoking-related knowledge, positive attitude, less smoking practices, and having a good plan to quit smoking.

## Figures and Tables

**Table 1:** General characteristics of the study population.

Variables		Males (3078)	Females (4920)	Total (7998)
Age ^a*∗∗*^		60.7±11.3	58.4±11.4	59.3±11.4
Education ^b*∗∗*^	No response	6(0.2)	8(0.2)	14 (0.2)
	< Middle schooling	1483(48.2)	3362 (68.3)	4845 (60.6)
	Middle schooling	1125 (36.5)	1201 (24.4)	2326 (29.1)
	High schooling	413 (13.4)	305 (6.2)	718 (9.0)
	College and above	51 (1.7)	44 (0.9)	95 (1.2)
Monthly income per person ^b^	<2000 RMB	2279 (74.0)	3722 (75.7)	6001 (75.0)
	2001-4000 RMB	567 (18.4)	889 (18.1)	1456 (18.2)
	>4000 RMB	232 (7.5)	309 (6.3)	541 (6.8)
Smoking status ^b*∗∗*^	No response	0 (0.0)	1(0.0)	1 (0.0)
	Yes	1732 (56.3)	61 (1.2)	1793 (22.4)
	No	1346 (43.7)	4858 (98.7)	6204 (77.6)
Second-hand smoking ^b^	No response	1(0.0)	2(0.0)	3 (0.0)
	Yes	1738 (56.5)	2861(58.2)	4599 (57.5)
	No	1339 (43.5)	2057 (41.8)	3396 (42.5)
Drinking alcohol ^b*∗∗*^	Never	1291 (41.9)	4440 (90.2)	5731 (71.7)
	Yes	1787 (58.1)	480 (9.8)	2265 (28.3)
BMI (Kg/m^2^) ^a*∗∗*^		24.6±3.5	25.1±3.6	24.9±3.6
BMI ^b*∗∗*^	<18.5	58 (1.9)	60 (1.2)	118 (1.5)
	18.5-23.9	1243 (41.3)	1807 (37.4)	3050(38.1)
	24.0-27.9	1269 (42.1)	2049 (42.4)	3318(41.5)
	≥28	442 (14.7)	912 (18.9)	1354(16.9)
WC (cm) ^a*∗∗*^		83.3±9.6	81.3±9.5	82.1±9.6
Central Obesity ^b*∗∗*^	Normal	2227(74.0)	2174 (44.2)	4451 (55.7)
	Waist ≥90 for males and waist ≥80 for females	801 (26.0)	2746 (56.8)	3547 (44.3)
WHR (WC/HR) ^a*∗∗*^		0.89±0.06	0.86±0.07	0.87±0.07
Abdominal Obesity ^b*∗∗*^	Normal	2251 (82.9)	599 (12.2)	3150 (39.4)
	Obese WHR> 0.94 males and >0.80 Females	527 (17.1)	4321 (87.8)	4848 (60.6)
s-KAP scores^∗∗^		11.7±3.6	13.1±5	12.5±3.7
Diet Scores^∗∗^		41.8±11.8	38.6±10.7	42.7±10.7

^a^=Independent t-test;  ^b^=Chi square test; ^∗∗^=*P<0.01*.

Data are expressed as mean±SD and N (%).

**Table 2:** Comparison of smoking-related KAP according to diet scores and anthropometric measurements.

	Low s-KAP(N=3987)	High s-KAP(N=4011)	P value
Diet scores	41.2±10.7 (40.8;41.5)	44.4±10.6 (43.7; 44.4)	P<0.001
BMI (Kg/m^2^)	24.8±3.7 (24.7; 24.9)	25.0±3.4 (24.9; 25.1)	0.015
Waist Circumference (cm)	82.5±9.7 (82.1, 82.8)	81.6±9.5 (81.3; 82.9)	P<0.001
WHR (WC/HC)	0.88±0.07 (0.878; 0.882)	0.87±0.06 (0.866, 0.870)	P<0.001

Independent t-tests were used for comparison.

Data are expressed as mean±SD (95 % CI).

**Table 3:** Multivariate linear regression analysis for the association of s-KAP scores and diet quality scores

Variables	Model 1	Model 2	Model 3
Whole population (N=7998)	0.341 (0.269, 0.414)^∗∗^	0.342 (0.270, 0. 414)^∗∗^	0.337 (0.265, 0.410)^∗∗^
Hypertension			
Yes (N=4656)	0.337 (0.240, 0.433)^∗∗^	0.338 (0.242, 0.434)^∗∗^	0.330(0.233, 0.426)^ ∗∗^
No (N=3342)	0.350 (0.240, 0.461)^∗∗^	0.351 (0.241, 0.461)^∗∗^	0.350 (0.240, 0.460)^ ∗∗^
Diabetes			
Yes (N=751)	0.530 (0.281, 0.779)^ ∗∗^	0.517 (0.270, 0.765)^∗∗^	0.515 (0.267, 0.763)^∗∗^
No (N=7247)	0.315 (0.240, 0.391)^∗∗^	0.316 (0.240, 0.391)^∗∗^	0.311 (0.236, 0.387)^∗∗^

Data as expressed as ß (95 % CI) values.

^∗^indicates *P<0.05;*^∗∗^indicates P<0.01

Model 1 is adjusted for age, gender, physical activity index, alcohol consumptions, educational levels, income, and BMI.

Model 2 is adjusted for age, gender, physical activity index, alcohol consumptions, educational levels, income, and WC.

Model 3 is adjusted for age, gender, physical activity index, alcohol consumptions, educational levels, income, and WHR.

## Data Availability

The data used to support the findings of this study are included within the article.

## Supplementary Materials

Supplementary Materials Smoking-related KAP questionnaire has been given in Supplementary Table 1. In the knowledge section, a total of eight questions were included regarding the relation of smoking (both active and passive) with diseases. In the attitude section, five items were included about the attitude of the participants towards smoking. Finally, in the practice section, four questions were included. The total maximum s-KAP scores were 20, including eight scores for knowledge, five scores for attitude, and seven scores for practices. Supplementary Table 2 describes the calculation of diet scores according to Chinese based dietary guidelines. Briefly, the CDGI components were including six adequacy components including grains, vegetables, fruits, nuts and soya bean products, milk & products, and seafood whereas four moderate products were included meat and poultry, oil, salt, and alcohol. For each component, 0-10 scores with the maximum total score of 100 scores for every individual. Supplementary Table 3 shows the difference between diet scores of SHS and smokers. There was a significant difference between the diet scores and s-KAP scores of SHS and smoking groups (P<0.01). Supplementary Table 4 shows the dietary intake according to s-KAP groups in the study population.. Cereals intake, alcohol consumption, sugar, and salt intake were higher (*P<0.05*) in the low s-KAP group. Whole grains, potato, seafood, nuts and soy products, poultry and meat, milk and products, and fruits intake of low s-KAP were significantly lower (*P<0.05*) as compared to the high s-KAP group. Supplementary Table 5 depicts the stratification correlation analysis of food groups' daily intake with s-KAP scores in the study population. For the whole population, a negative correlation (*P*<0.01) of s-KAP scores was observed with cereals (rice, wheat flour, noodles) intake (r=-0.178) and alcohol intake (r=-0.161). While a positive correlation (*P<0.01*) with whole grain (r=0.157), potato (r=0.125), seafood (r=0.1), and milk and products (r=0.157) with s-KAP scores was observed. Particular in females, cereals was having negative association while potato, whole grains, eggs, sea food, milk, meat and poultry, and nuts and soya had positive association with s-KAP scores (r>0.1, P<0.05). Supplementary Table 6 reveals the multivariate linear regression analysis for the association nutritional status and diet quality scores. There was no significant association (P>0.05) of nutritional status based on BMI, WC and WHR with s-KAP scores after adjustment for age, gender, physical activity index, alcohol consumptions, smoking, educational levels, income, and s-KAP scores. Similarly, in stratification analysis for both hypertension and diabetes, the association of nutritional status with s-KAP scores was also not significant after adjustment for covariates (P>0.05). Supplementary Table 7 shows the multivariate linear regression analysis for the association smoking and diet quality scores. Smoking was significantly negatively associated with diet scores after adjustment for covariates (P<0.05). Similarly, in stratification analysis for both hypertension and diabetes, the association of smoking with diet scores remains significant after adjustment for covariates (P<0.05). Supplementary Table 8 reveals the multivariate linear regression analysis for the association SHS and diet quality scores. There was no significant association (P>0.05) of SHS with diet scores after adjustment for covariates. Similarly, in stratification analysis for both hypertension and diabetes, the association of nutritional status with s-KAP scores was also not significant after adjustment for covariates (P>0.05).

## References

[1] Li Q., Hsia J., Yang G. (2011). Prevalence of smoking in China in 2010. *The New England Journal of Medicine*.

[2] Gu D., Kelly T. N., Wu X. (2009). Mortality attributable to smoking in China. *The New England Journal of Medicine*.

[3] Yang L., Chu T. K., Lian J. (2018). Risk factors of chronic kidney diseases in Chinese adults with type 2 diabetes. *Scientific Reports*.

[4] Lim J., Lee Y., Shin S. (2018). An association between diet quality index for Koreans (DQI-K) and total mortality in health examinees gem (HEXA-G) study. *Nutrition Research and Practice*.

[5] Alkerwi A., Baydarlioglu B., Sauvageot N. (2017). Smoking status is inversely associated with overall diet quality: Findings from the ORISCAV-LUX study. *Clinical Nutrition*.

[6] Adriouch S., Lelong H., Kesse-Guyot E. (2017). Compliance with nutritional and lifestyle recommendations in 13,000 patients with a cardiometabolic disease from the nutrinet-santé study. *Nutrients*.

[7] ul Haq I., Mariyam Z., Li M. (2018). A comparative study of nutritional status, knowledge attitude and practices (KAP) and dietary intake between international and chinese students in Nanjing, China. *International Journal of Environmental Research and Public Health*.

[8] Alkerwi A., Sauvageot N., Malan L., Shivappa N., Hébert J. R. (2015). Association between nutritional awareness and diet quality: evidence from the observation of cardiovascular risk factors in Luxembourg (ORISCAV-LUX) study. *Nutrients*.

[9] Geaney F., Fitzgerald S., Harrington J., Kelly C., Greiner B., Perry I. (2015). Nutrition knowledge, diet quality and hypertension in a working population. *Preventive Medicine Reports*.

[10] Raatz S. K., Jahns L., Johnson L. K. (2017). Smokers report lower intake of key nutrients than nonsmokers, yet both fall short of meeting recommended intakes. *Nutrition Research*.

[11] Chan S. S., Sarna L., Wong D. C., Lam T. (2007). Nurses' tobacco-related knowledge, attitudes, and practice in four major cities in China. *Journal of Nursing Scholarship*.

[12] Yan J., Xiao S., Ouyang D., Jiang D., He C., Yi S. (2008). Smoking behavior, knowledge, attitudes and practice among health care providers in Changsha city, China. *Nicotine & Tobacco Research*.

[13] Xu X., Liu L., Sharma M., Zhao Y. (2015). Smoking-related knowledge, attitudes, behaviors, smoking cessation idea and education level among young adult male smokers in Chongqing, China. *International Journal of Environmental Research and Public Health*.

[14] Iredale J. M., Clare P. J., Courtney R. J. (2016). Associations between behavioural risk factors and smoking, heavy smoking and future smoking among an Australian population-based sample. *Preventive Medicine*.

[15] Schoufour J. D., de Jonge E. A., Kiefte-de Jong J. C. (2018). Socio-economic indicators and diet quality in an older population. *Maturitas*.

[16] Lin C.-T. J., Gao Z., Lee J.-Y. (2013). Associations between self-reported weight management methods with diet quality as measured by the Healthy Eating Index-2005. *Preventive Medicine*.

[17] Martin M. A., Van Hook J. L., Quiros S. (2015). Is socioeconomic incorporation associated with a healthier diet? Dietary patterns among Mexican-origin children in the United States. *Social Science & Medicine*.

[18] Ahola A. J., Saraheimo M., Freese R., Forsblom C., Mäkimattila S., Groop P. (2017). Association between adherence to dietary recommendations and high-sensitivity C-reactive protein level in type 1 diabetes. *Diabetes Research and Clinical Practice*.

[19] Wang Z., Siega-Riz A., Gordon-Larsen P. (2018). Diet quality and its association with type 2 diabetes and major cardiometabolic risk factors among adults in China. *Nutrition, Metabolism & Cardiovascular Diseases*.

[20] Zhou B. F. (2002). Cooperative meta-analysis group of the working group on obesity in C: predictive values of body mass index and waist circumference for risk factors of certain related diseases in Chinese adults--study on optimal cut-off points of body mass index and waist circumference in Chinese adults. *Biomedical and Environmental Sciences*.

[21] Yang W., Gao X., Zhang X. (2018). Impact of interactions among metabolic syndrome components on the development of cardiovascular disease among Kazakhs in Xinjiang. *PLoS ONE*.

[22] Tsigos C., Hainer V., Basdevant A. (2008). Management of obesity in adults: European clinical practice guidelines. *Obesity Facts*.

[23] Wientzek A., Vigl M., Steindorf K. (2014). The improved physical activity index for measuring physical activity in EPIC Germany. *PLoS ONE*.

[24] Pavičić Žeželj S., Kenđel Jovanović G., Krešić G. (2019). The association between the Mediterranean diet and high physical activity among the working population in Croatia. *Medycyna Pracy*.

[25] Backholer K., Spencer E., Gearon E. (2016). The association between socio-economic position and diet quality in Australian adults. *Public Health Nutrition*.

[26] Nicolaou M., van Dam R. M., Stronks K. (2006). Acculturation and education level in relation to quality of the diet: a study of Surinamese South Asian and Afro-Caribbean residents of the Netherlands. *Journal of Human Nutrition and Dietetics*.

[27] Bøgh-Sørensen L., Biltoft-Jensen A., Groth M. V., Matthiessen J., Fagt S., Hels O. (2009). Association between alcohol intake and diet quality. *Ugeskrift for Læger*.

[28] Fallaize R., Livingstone K., Celis-Morales C. (2018). Association between diet-quality scores, adiposity, total cholesterol and markers of nutritional status in european adults: findings from the Food4Me study. *Nutrients*.

[29] Demaio A. R., Nehme J., Otgontuya D., Meyrowitsch D. W., Enkhtuya P. (2014). Tobacco smoking in Mongolia: findings of a national knowledge, attitudes and practices study. *BMC Public Health*.

[30] Saw A., Tang H., Tsoh J. Y., Chen M. S., Tong E. K. (2017). Non-smoker assertive behaviour against smoke exposure: Chinese and Korean American non-smokers. *Drug and Alcohol Review*.

[31] Poland B. D., Cohen J. E., Ashley M. J. (2000). Heterogeneity among smokers and non-smokers in attitudes and behaviour regarding smoking and smoking restrictions. *Tobacco Control*.

[32] Yang J., Hammond D., Driezen P., Fong G. T., Jiang Y. (2010). Health knowledge and perception of risks among chinese smokers and non-smokers: findings from the wave 1 ITC china survey. *Tobacco Control*.

[33] Frosch Z. A., Dierker L. C., Rose J. S., Waldinger R. J. (2009). Smoking trajectories, health, and mortality across the adult lifespan. *Addictive Behaviors*.

[34] Cheah Y. K., Naidu B. M. (2012). Exploring factors influencing smoking behaviour in Malaysia. *Asian Pacific Journal of Cancer Prevention*.

[35] Carreras-Torres R., Johansson M., Haycock P. C. (2018). Role of obesity in smoking behaviour: Mendelian randomisation study in UK Biobank. *BMJ*.

[36] Efendi V., Özalevli S., NAZ İ., Kılınç O. (2018). The effects of smoking on body composition, pulmonary function, physical activity and health-related quality of life among healthy women. *Tüberküloz ve Toraks*.

[37] Sneve M., Jorde R. (2008). Cross-sectional study on the relationship between body mass index and smoking, and longitudinal changes in body mass index in relation to change in smoking status: the Tromso Study. *Scandinavian Journal of Public Health*.

[38] Son S., Ro Y., Hyun H., Lee H., Song K. (2014). A comparative study on dietary behavior, nutritional knowledge and life stress between Korean and Chinese female high school students. *Nutrition Research and Practice*.

[39] Larson N. I., Story M., Perry C. L., Neumark-Sztainer D., Hannan P. J. (2007). Are diet and physical activity patterns related to cigarette smoking in adolescents? Findings from project EAT. *Preventing Chronic Disease*.

[40] MacLean R. R., Cowan A., Vernarelli J. A. (2018). More to gain: Dietary energy density is related to smoking status in US adults. *BMC Public Health*.

