# Supplementary material for: Association of Smoking-Related Knowledge, Attitude, and Practices (KAP) with Nutritional Status and Diet Quality: A Cross-Sectional Study in China
Source: Biomed Res Int. 2019 Aug 21;2019:5897478. doi: 10.1155/2019/5897478 (PMC6720361; doi:10.1155/2019/5897478)
Supplement: Supplementary Materials — Smoking-related KAP questionnaire has been given in Supplementary Table 1. In the knowledge section, a total of eight questions were included regarding the relation of smoking (both active and passive) with diseases. In the attitude section, five items were included about the attitude of the participants towards smoking. Finally, in the practice section, four questions were included. The total maximum s-KAP scores were 20, including eight scores for knowledge, five scores for attitude, and seven scores for practices. Supplementary Table 2 describes the calculation of diet scores according to Chinese based dietary guidelines. Briefly, the CDGI components were including six adequacy components including grains, vegetables, fruits, nuts and soya bean products, milk & products, and seafood whereas four moderate products were included meat and poultry, oil, salt, and alcohol. For each component, 0-10 scores with the maximum total score of 100 scores for every individual. Supplementary Table 3 shows the difference between diet scores of SHS and smokers. There was a significant difference between the diet scores and s-KAP scores of SHS and smoking groups (P<0.01). Supplementary Table 4 shows the dietary intake according to s-KAP groups in the study population.. Cereals intake, alcohol consumption, sugar, and salt intake were higher (P<0.05) in the low s-KAP group. Whole grains, potato, seafood, nuts and soy products, poultry and meat, milk and products, and fruits intake of low s-KAP were significantly lower (P<0.05) as compared to the high s-KAP group. Supplementary Table 5 depicts the stratification correlation analysis of food groups' daily intake with s-KAP scores in the study population. For the whole population, a negative correlation (P<0.01) of s-KAP scores was observed with cereals (rice, wheat flour, noodles) intake (r=-0.178) and alcohol intake (r=-0.161). While a positive correlation (P<0.01) with whole grain (r=0.157), potato (r=0.125), seafood (r=0.1), and mi [file 5897478.f1.docx]

**Supplementary Materials**

Smoking-related KAP questionnaire has been given in **Supplementary table 1**. In the knowledge section, a total of eight questions were included regarding the relation of smoking (both active and passive) with diseases. In the attitude section, five items were included about the attitude of the participants towards smoking. Finally, in the practice section, four questions were included. The total maximum s-KAP scores were 20, including eight scores for knowledge, five scores for attitude, and seven scores for practices.

**Supplementary table 2** describes the calculation of diet scores according to Chinese based dietary guidelines. Briefly, the CDGI components were including six adequacy components including grains, vegetables, fruits, nuts and soya bean products, milk & products, and seafood whereas four moderate products were included meat and poultry, oil, salt, and alcohol. For each component, 0-10 scores with the maximum total score of 100 scores for every individual.

**Supplementary table 3** shows the difference between diet scores of SHS and smokers. There was a significant difference between the diet scores and s-KAP scores of SHS and smoking groups (P<0.01).

**Supplementary table 4** shows the dietary intake according to s-KAP groups in the study population. . Cereals intake, alcohol consumption, sugar, and salt intake were higher (*P<0.05*) in the low s-KAP group. Whole grains, potato, seafood, nuts and soy products, poultry and meat, milk and products, and fruits intake of low s-KAP were significantly lower (*P<0.05*) as compared to the high s-KAP group.

**Supplementary table 5** depicts the stratification correlation analysis of food groups’ daily intake with s-KAP scores in the study population. For the whole population, a negative correlation (*P*<0.01) of s-KAP scores was observed with cereals (rice, wheat flour, noodles) intake (r=-0.178) and alcohol intake (r=-0.161). While a positive correlation (*P<0.01*) with whole grain (r=0.157), potato (r=0.125), seafood (r=0.1), and milk and products (r=0.157) with s-KAP scores was observed. Particular in females, cereals was having negative association while potato, whole grains, eggs, sea food, milk, meat and poultry, and nuts and soya had positive association with s-KAP scores (r>0.1, P<0.05).

**Supplementary table 6** reveals the multivariate linear regression analysis for the association nutritional status and diet quality scores. There was no significant association (P>0.05) of nutritional status based on BMI, WC and WHR with s-KAP scores after adjustment for age, gender, physical activity index, alcohol consumptions, smoking, educational levels, income, and s-KAP scores. Similarly, in stratification analysis for both hypertension and diabetes, the association of nutritional status with s-KAP scores was also not significant after adjustment for covariates (P>0.05).

**Supplementary table 7** shows the multivariate linear regression analysis for the association smoking and diet quality scores. Smoking was significantly negatively associated with diet scores after adjustment for covariates (P<0.05). Similarly, in stratification analysis for both hypertension and diabetes, the association of smoking with diet scores remains significant after adjustment for covariates (P<0.05).

**Supplementary table 8** reveals the multivariate linear regression analysis for the association SHS and diet quality scores. There was no significant association (P>0.05) of SHS with diet scores after adjustment for covariates. Similarly, in stratification analysis for both hypertension and diabetes, the association of nutritional status with s-KAP scores was also not significant after adjustment for covariates (P>0.05).

Supplementary table 1 Smoking-related KAP questionnaire and scores

| Smoking KAP items | Score | Maximum scores |
| --- | --- | --- |
| Smoke-related knowledge   1. Active smoking leads to cerebral apoplexy 2. Active smoking leads to lungs cancer 3. Active smoking leads to CVD 4. Active smoking leads to cataract eye disease 5. Active smoking leads to abortion or miscarriage 6. Active smoking leads to low birth weight infants 7. Passive smoking leads to lungs cancer 8. Passive smoking leads to CVD | For correct answer one score for each question | 8 |
| Attitude   1. The damage of low tar cigarette is smaller than the common cigarette 2. Every cigarette is harmful to the health of smokers 3. Tobacco can be addictive 4. Smoking should be prohibited in public places 5. The government should take strong action against smoking | For a positive attitude for each question 1 score | 5 |
| Practices   1. Smoking score 2. Second-hand exposure for more than 15 minutes 3. Stopped smoking in last one year 4. Quit smoking plan | 1. Smoking=0, Occasionally=1, No smoking=2  2. Seldom=1, Exposed=0  3. Non-smokers didn't touch tobacco and stopped smoking=2, No=0  4. Non-smoker=2, Next month=1, Next year=0.5, No plan and not sure=0 | 7 |
| Total KAP scores |  | 20 |

Supplementary table 2 The principals for calculating the Diet score according to Chinese dietary guidelines Index

| Food groups | Minimum score’s criterion | Minimum score’s criterion (0) | Maximum scores (100) |
| --- | --- | --- | --- |
| Grains | 0 g/d | ≥75 g/d | 10 |
| Total vegetables including dark leafy vegetables | 0 g/d | ≥300-500 g/d | 10 |
| Fruits | 0/g/d | 200-400 g/d | 10 |
| Nuts and soya products | 0/g/d | 30-50 g/d | 10 |
| Dairy and products | 0/g/d | ≥300 g/d | 10 |
| Seafood | 0/g/d | 50-100 g/d | 10 |
| Meat and poultry | ≥(50-75) g/d | 0/g/d | 10 |
| Cooking oil | ≥2(25-30) g/d | <(25-30) g/d | 10 |
| Salt intake | ≥ 12 g/d | < 6 g/d | 10 |
| Alcohol consumptions | Male ≥ 50 g/d  Female ≥ 30 g/d | Male <25 g/d  Female <15 g/d | 10 |

Supplementary table 3 Difference between diet scores of SHS and smokers

| Score | SHS | Smokers | t/Z | P value |
| --- | --- | --- | --- | --- |
| Diet scores ^a^ | 42.74±10.9 | 40.6±10.5 | 6.77 | <0.001 |
| s-KAP scores^b^ | 12.0 (10.0, 15.0) | 10 (7.0, 12.0) | -22.6 | <0.001 |

^a^=t test, ^b^ = Mann whitney test

Supplementary table 4 Dietary intake according to s-KAP in the study population

| Dietary intake | Low s-KAP (N=3987) | High s-KAP (N=4011) | Z | P value |
| --- | --- | --- | --- | --- |
| Cereals (g/day) | 341.8, 375.0 (125) | 310.0, 250.0 (125.0) | -13.2 | P<0.001 |
| Whole grains(g/day) | 12.5, 0.0 (0.0) | 22.8, 0.0 (10.5) | -12.2 | P<0.001 |
| Potato (g/day) | 20.5, 7.0 (21.0) | 26.9, 10.5 (50.0) | -10.7 | P<0.001 |
| Eggs (1 medium) | 0.62, 0.61 (0.9) | 0.67, 0.61 (0.8) | -4.7 | P<0.001 |
| Sea food (g/day) | 40.5, 30.5 (54.0) | 48.1, 61.0 (54.0) | -8.9 | P<0.001 |
| Poultry and meat | 72.5, 61.0 (86.0) | 78.9, 64.5 (76.2) | -3.9 | P<0.001 |
| Milk and products (g/day) | 24.0, 0.0 (17.5) | 25.3, 0.0 (17.5) | -6.6 | P<0.001 |
| Nuts and Soya products (g/day) | 37.5, 30.5 (54.0) | 46.6, 31.5 (50.5) | -3.2 | 0.002 |
| Fruit (g/day) | 44.9, 32.0 (64.0) | 51.7, 32.0 (96.0) | -6.8 | P<0.001 |
| Vegetables (g/day) | 133.7, 150.0 (50.0) | 134.8, 150.0 (50.0) | -0.6 | 0.510 |
| Alcohol (g/d) | 20.7, 0.0 (38.0) | 12.1, 0.0 (53.0) | -12.0 | P<0.001 |
| Oil (g/day) | 39.5, 35.0 (22.7) | 39.1, 35.0 (21.4) | -0.18 | 0.851 |
| Salt (g/day) | 9.8, 8.3 (5.8) | 9.0, 8.3 (3.8) | -5.2 | P<0.001 |
| Sugar (g/day) | 3.5, 2.5 (4.2) | 2.9, 2.2 (4.2) | -1.0 | 0.287 |
| Pickle (g/day) | 6.9, 4.1 (8.3) | 5.9, 3.5 (8.3) | -1.4 | 0.137 |
| Tofu (g/day) | 0.7, 0.0 (0.0) | 0.5, 0.0 (0.0) | -5.2 | P<0.001 |

Mann-Whitney tests were used for comparison.

Data are expressed as mean, median (IQR).

Supplementary table 5 Correlation analysis of food groups daily intake with s-KAP scores

| Variables | Whole | Male | Female |
| --- | --- | --- | --- |
| Cereals (g/day) | -0.178^**^ | -0.084^**^ | -0.133^**^ |
| Whole grains (g/day) | 0.157^**^ | 0.148^**^ | 0.123^**^ |
| Potato (g/day) | 0.125^**^ | 0.082^**^ | 0.125^**^ |
| Eggs (1) | 0.040^**^ | 0.027 | 0.105^**^ |
| Sea food (g/day) | 0.10^**^ | 0.082^**^ | 0.149^**^ |
| Meat and poultry(g/day) | 0.039^**^ | 0.018 | 0.114^**^ |
| Milk (g/day) | 0.10^**^ | 0.072^**^ | 0.10^**^ |
| Nuts and Soya products (g/day) | 0.040^**^ | -0.002 | 0.10^**^ |
| Fruit (g/day) | 0.081^**^ | 0.102^**^ | .032^*^ |
| Vegetables (g/day) | 0.008 | 0.010 | .003 |
| Alcohol (g/d) | -0.161^**^ | -0.081^**^ | -0.007 |
| Oil (g/day) | 0.003 | -0.004 | 0.005 |
| Salt (g/day) | -0.066^**^ | -0.033 | -0.072^**^ |
| Sugar (g/day) | 0.016 | 0.000 | 0.030^*^ |
| Pickle (g/day) | -.019 | 0.014 | -0.040^**^ |
| Tofu (g/day) | 0.069^**^ | 0.070^**^ | 0.077^**^ |
| Sauce (g/day) | 0.059^**^ | 0.051^**^ | 0.067^**^ |

^**^=P<0.01,

Partial correlation was used for correlation

Supplementary table 6 Multivariate linear regression analysis for the association of nutritional status and s-KAP scores

| Variables | BMI | WC | WHR |
| --- | --- | --- | --- |
| Whole population (N=7998) | -0.004 (-0.022, -0.030) | 0.021 (-0.047, 0.90) | -0.003 (-0.008, 0.002) |
| Hypertension |  |  |  |
| Yes (N=4656) | 0.008 (-0.027, 0.044) | 0.033 (-0.057, 0.123) | -0.003 (-0.010, 0.003) |
| No (N=3342) | 0.001 (-0.035, 0.037) | 0.013 (-0.86, 0.113) | -0.002 (-0.009, 0.005) |
| Diabetes |  |  |  |
| Yes (N=751) | -0.048 (-0.141, 0.044) | -0.060(-0.283, 0.163) | -0.008 (-0.022, 0.006) |
| No (N=7247) | 0.008 (-0.019, 0.035) | 0.028 (-0.043, 0. 100) | -0.001 (-0.006, 0.004) |

Data are expressed as ß (95 % CI) values of linear regression.

All models are adjusted for age, gender, physical activity index, alcohol consumptions, smoking, educational levels, income, and diet scores.

Supplementary table 7 Multivariate linear regression analysis for the association of smoking and diet quality scores

| Variables | Model 1 | Model 2 | Model 3 |
| --- | --- | --- | --- |
| Whole population (N=7998) | -1.592 (-2.002, -1.181) ^**^ | -1.596 (-2.005, -1.186)^**^ | -1.564 (-1/974, -1.155)^**^ |
| Hypertension |  |  |  |
| Yes (N=4656) | -1.726 (-2.249, -1.204)^**^ | -1.727 (-2.248, -1.206)^**^ | -1.688 (-2.209, -1.167)^**^ |
| No (N=3342) | -1.378 (-2.051, -0.706)^**^ | -1.384 (-2.056, -0.713)^**^ | -1.371 (-2.043, -0.699)^**^ |
| Diabetes |  |  |  |
| Yes (N=751) | -0.967 (-2.406, -0.473) | -0.861 (-2.295, 0.573) | -0.864 (-2.299, 0.570) |
| No (N=7247) | -1.637 (-2.064, -1.210)^**^ | -1.644 (-2.071, -1.218)^**^ | -1.611 (-2.037, -1.185)^**^ |

Data are expressed as ß (95 % CI) values of linear regression.

^**^indicates P<0.01

Model 1: Adjusted for age, gender, physical activity index, alcohol consumptions, educational levels, income and BMI.

Model 2: Adjusted for age, gender, physical activity index, alcohol consumptions, educational levels, income and WC.

Model 3: Adjusted for age, gender, physical activity index, alcohol consumptions, educational levels, income and WHR.

Supplementary table8 Multivariate linear regression analysis for the association of SHS and diet quality scores

| Variables | Model 1 | Model 2 | Model 3 |
| --- | --- | --- | --- |
| Whole population (N=7998) | 0.329 (-0.180, 0.838) | 0.361 (-0.147, 0.870) | 0.335 (-0.173, 0.844) |
| Hypertension |  |  |  |
| Yes (N=4656) | 0.394 (-0.301, 1.089) | 0.425(-0.269, 1.119) | 0.410 (-0.284, 1.104) |
| No (N=3342) | 0.263 (-0.486, 1.012) | 0.282 (-0.467, 1.031) | 0.264 (-0.485, 1.014) |
| Diabetes |  |  |  |
| Yes (N=751) | 0.128 (-1.650, 1.907) | 0.268 (-1.503, 2.038) | 0.255 (-1.516, 2.026) |
| No (N=7247) | 0.357 (0.173, 0.887) | 0.378 (0.152, 0.907) | 0.356 (-0.174, 0.855) |

Data are expressed as ß (95 % CI) values of linear regression.

**indicates P<0.01

Model 1: Adjusted for age, gender, physical activity index, alcohol consumptions, educational levels, income and BMI.

Model 2: Adjusted for age, gender, physical activity index, alcohol consumptions, educational levels, income and WC.

Model 3: Adjusted for age, gender, physical activity index, alcohol consumptions, educational levels, income and WHR.
